# Complete Genome Sequence of a Marine Bacterium, *Pseudomonas pseudoalcaligenes* Strain S1, with High Mercury Resistance and Bioaccumulation Capacity

**DOI:** 10.1128/genomeA.00381-16

**Published:** 2016-05-19

**Authors:** Bing Liu, Chao Bian, Huiwei Huang, Zhiwei Yin, Qiong Shi, Xu Deng

**Affiliations:** a Shenzhen Key Laboratory of Marine Bioresources and Ecology, College of Life Science, Shenzhen University, Shenzhen, Guangdong, China; b State Key Laboratory of Agriculture Genomics, Shenzhen Key Laboratory of Marine Genomics, BGI, Shenzhen, Guangdong, China

## Abstract

*Pseudomonas pseudoalcaligenes* S1, a marine bacterium, exhibited strong resistance to a high concentration of Hg^2+^ and remarkable Hg^2+^ bioaccumulation capacity. Here, we report the 6.9-Mb genome sequence of *P. pseudoalcaligenes* S1, which may help clarify its phylogenetic status and provide further understanding of the mechanisms of mercury bioremediation in a marine environment.

## GENOME ANNOUNCEMENT

Bacteria have been used for bioremediation because they have a high capability for heavy metal uptake under a wide range of external conditions, such as high salinity and the presence of a variety of ions in the marine environment (1–3). On 6 October 2010, we isolated *P. pseudoalcaligenes* S1 (CGMCC 7.206) from seawater and marine sediment samples in a mangrove conservation district of Shenzhen Bay, China (113.944°E, 22.486°N). *P. pseudoalcaligenes* S1 was preliminarily investigated by us (4) for its bioaccumulation and attractive mercury resistance which has the highest value reported so far (5, 6). It has generally been accepted that the mercury resistance of microorganisms depends on the intracellular expression of MerA, a mercuric reductase conferring cells to reduce Hg^2+^ to Hg^0^ (7). In this case, microbial cells will not exhibit high Hg^2+^ accumulation capacity, because Hg^0^ is subject to volatilization into the air. However, the high Hg^2+^ accumulation capacity of *P. pseudoalcaligenes* S1 suggested that the mercuric reduction strategy cannot completely explain its tolerance of high Hg^2+^ concentrations. Therefore, we hypothesize a novel mechanism in this marine bacterium for both strong Hg^2+^ tolerance and high Hg^2+^ bioaccumulation. In our current paper, we performed whole-genome sequencing of *P. pseudoalcaligenes* S1 in an attempt to provide the genetic basis for further understanding the mechanisms of mercury bioremediation and resistance.

The *P. pseudoalcaligenes* S1 genome was sequenced by an Illumina HiSeq 2000 platform with the high deep shotgun strategy (8). Two independent libraries with insert sizes of 500 bp and 6,000 bp were constructed using the standard protocol from Illumina (San Diego, CA, USA). We obtained 1.18 Gb of raw data. SOAP*denovo*2 (version 2.04.4) (9) with optimized parameters (pregraph, K 35 –d 1; contig, M 0; scaff, F –b 1.5 –p 16) was subsequently employed to assemble the genome sequences, and Gapcloser1.10 was used to fill the gaps in the scaffolds, finally resulting in a 6.9-Mb assembly (62.5% G+C content). The generated assembly is composed of 131 scaffolds and 133 contigs, with *N*_50_ values of 1.4 Mb. All the assembled data were deposited in the NCBI nucleotide sequence database.

Protein-coding genes were predicted using the NCBI Prokaryotic Genome Annotation Pipeline, which is designed to annotate bacterial and archaeal genomes. Finally, we obtained 6,500 genes, 5,779 protein-coding genes, 78 tRNAs, 8 rRNAs, and 1 noncoding RNA.

Genes for mercuric regulation (*merR*), mercuric transport and binding (*merT* and *merP*), and metal ion efflux pump (CzcA) were identified from the *P. pseudoalcaligenes* S1 complete genome. They may be responsible for the adaptation of mercury contamination through transmembrane transportation and active efflux (10–13), therefore, they may help practice the function of high mercury resistance and accumulation in the marine bacterium *P. pseudoalcaligenes* S1. In addition, the gene for mercuric reduction (*merA*) was also predicted from our transcriptome data; however, its function remains a mystery.

### Nucleotide sequence accession numbers.

This whole-genome shotgun project has been deposited in DDBJ/EMBL/GenBank under the accession no. JTFL00000000. The version described in this paper is the first version, JTFL01000000.
